# Differentially expressed microRNAs in bone marrow mesenchymal stem cell-derived microvesicles in young and older rats and their effect on tumor growth factor-β1-mediated epithelial-mesenchymal transition in HK2 cells

**DOI:** 10.1186/s13287-015-0179-x

**Published:** 2015-09-28

**Authors:** Yan Wang, Bo Fu, Xuefeng Sun, Diangeng Li, Qi Huang, Weihong Zhao, Xiangmei Chen

**Affiliations:** Department of Geriatrics, the First Affiliated Hospital of Nanjing Medical University, 300 Guangzhou Road, Nanjing, Jiangsu 210029 China; Department of Nephrology, Chinese PLA General Hospital, Chinese PLA Institute of Nephrology, State Key Laboratory of Kidney Diseases, National Clinical Research Center for Kidney Diseases, 28 Fuxing Road, Beijing, 100853 China

## Abstract

**Introduction:**

The prevalence of renal fibrosis is higher in older than in younger individuals. Through paracrine activity, bone marrow mesenchymal stem cell-derived microvesicles (BM-MSC-MVs) influence the process of renal fibrosis. Differences in microRNA (miRNA) expression of BM-MSC-MVs that correlate with the age of the subjects and the correlation between miRNA expression and the process of renal fibrosis have not been established. The present study aimed to analyze differences in miRNA expression of BM-MSC-MVs between young or older rats and its influence on tumor growth factor-beta 1 (TGF-β1)-mediated epithelial-mesenchymal transition (EMT) of HK2 cells to explore the causes of renal fibrosis in aged tissues.

**Methods:**

miRCURY LNA Array (version 18.0) was used to identify differentially expressed miRNAs in BM-MSC-MVs of 3- and 24-month-old Fisher344 rats. Reverse transcription-polymerase chain reaction was used to verify miRNA levels in BM-MSC-MVs and in the serum of rats. A TGF-β1-mediated EMT model was used to study the effects of BM-MSC-MVs and differentially expressed miRNAs on EMT.

**Results:**

BM-MSCs from older rats showed more severe aging phenotypes compared with those of young rats. In addition, the growth rate and cell migration of BM-MSCs derived from older rats were significantly reduced. In secreted BM-MSC-MVs, the expression of miR-344a, miR-133b-3p, miR-294, miR-423-3p, and miR-872-3p was significantly downregulated in older rats than in younger rats (*P* < 0.05), and the serum level of these miRNAs exhibited the same patterns. Intervention using BM-MSC-MVs resulted in the weakening of TGF-β1-mediated EMT in the aged rats. MiR-344a, miR-133b-3p, and miR-294 affected TGF-β1-mediated EMT in HK2 cells. Among these, miR-133b-3p and miR-294 significantly inhibited TGF-β1-mediated EMT in HK2 cells (*P* < 0.05).

**Conclusions:**

In older rats, the inhibitory effect of BM-MSC-MVs on TGF-β1-mediated HK2 cell EMT was weaker than that observed in younger rats. In addition, miR-133b-3p and miR-294, which were downregulated in BM-MSC-MVs of older rats, remarkably inhibited TGF-β1-mediated EMT in HK2 cells, suggesting that these may play a role in the fibrosis of aging renal tissues.

**Electronic supplementary material:**

The online version of this article (doi:10.1186/s13287-015-0179-x) contains supplementary material, which is available to authorized users.

## Introduction

Chronic kidney disease (CKD) affects older people at a higher rate compared with younger subjects. According to systematic reviews and analyses, the incidence of CKD has significantly increased with the improvement on the life span of the general population [1]. Based on Chinese epidemiological surveys on CKD, the average age of patients with stage 3 CKD or higher is 63.6 years old. With an increase of 10 years in age, the risk of epidermal growth factor receptor (eGFR) is less than 60 ml/min per 1.73 m^2^, which represents the renal glomerular filtration rate, increasing by 1.74-fold [2]. According to the US National Health and Nutrition Examination Survey, the incidences of phase 1 or 2 CKD were 2 %–3 % among people within the age of 20 to 39 years and 9 %–10 % among people above 70 years old, whereas those of phase 3 or 4 CKD were 0.2 %–0.7 % among people with the age range of 20 to 39 years old and 27.8 %–37.8 % among people above 70 years old [3]. Tubulointerstitial fibrosis plays an important role in the course of CKD and is a typical characteristic of aged kidney tissues [2, 3]. Tumor growth factor-beta 1 (TGF-β1) is an important growth factor that induces epithelial-to-mesenchymal transition (EMT) and promotes tubulointerstitial fibrosis [4].

Bone marrow mesenchymal stem cells (BM-MSCs) are a pluripotent cell population that can differentiate into various cell types such as fat, bone, muscle, and skin cells as well as show significant therapeutic effects against many diseases [5–7]. It has been recently found that BM-MSCs change accordingly with the age of an individual [8–10]. With aging, BM-MSCs decrease in number [11, 12], and the functional factors they secrete may also change [12–14]. Microvesicles (MVs) are small-particle exosomes that are secreted by BM-MSCs and contain a number of bioactive substances such as proteins, lipids, mRNA, and microRNA (miRNA). These also have good therapeutic effects against acute and chronic diseases [15–17]. miRNAs are important genetic regulatory factors of BM-MSC-MVs that identify target mRNAs through base pairing. Moreover, based on the degree of complementarity, miRNAs can guide silencing complexes to degrade target mRNAs or downregulate the expression of mRNAs. Previous studies have shown that miRNAs are closely linked to the occurrence of diseases and the aging process [18–20]. However, information on differences in miRNA expression in BM-MSC-MVs between young and elder individuals and whether these differences can further promote renal fibrosis and aging of kidney is unknown. The present study aimed to analyze differences in miRNA expression of BM-MSC-MVs between young or elder rats as well as the effect of differentially expressed miRNAs on TGF-β1-mediated EMT of HK2 cells in relation to the pathogenesis of renal fibrosis in aged tissues.

## Methods

### Cell culture and identification of BM-MSCs

Animal welfare and experimental procedures were all carried out in accordance with the Guide for the Care and Use of Laboratory Animals (Ministry of Science and Technology of China, 2006) and were approved by the animal ethics committee of the General Hospital of the People’s Liberation Army. BM-MSCs were extracted from 3- and 24-month-old Fisher344 rats [16]. The isolated primary BM-MSCs were cultured in complete medium (Cyagen Bioscience Inc., Santa Clara, CA, USA). After the cells were passaged to P2, BM-MSCs were identified by flow cytometry by using cell surface markers, CD44, CD105, CD146, and CD45 (BioLegend Inc., San Diego, CA, USA).

### Proliferation, migration, and differentiation of BM-MSCs

#### BM-MSC proliferation assay

Both P2 BM-MSCs from young and elder rats were counted after trypsinization by using 0.25 % trypsin (Corning, Corning, NY, USA), and then approximately 10,000 cells were resuspended in 100 μl of media and seeded onto each well of a 96-well plate. After culturing for 4 days, each well was supplemented with 10 μl of a cck-8 solution (Dojindo Inst. Biotech, Shanghai, China). The plate was cultured for 3 h in an incubator. The absorbance at a wavelength of 450 nm was measured by using a microplate reader. Each group had five replicate experimental sets.

#### BM-MSC migration assay

Polycarbonate membrane transwell inserts (8 μm) were used (Corning). Before cell inoculation, upper transwell chambers were equilibrated for 30 min by using serum-free RPMI 1640 medium (Corning). Then in the uncoated upper chambers, 1 × 10^5^ P2 BM-MSCs, which had been resuspended in serum-free RPMI 1640 medium, were inoculated. In the lower chambers, normal medium containing 10 % serum (Cyagen Bioscience Inc.) was added. After incubating for 24 h, cells that had not migrated across the polycarbonate membranes were gently wiped off. Then the cells on the underside of the membranes were fixed for 30 min with 4 % formaldehyde and stained with crystal violet (Beyotime Institute of Biotechnology, Jiangsu, China). Cells that migrated across the polycarbonate membrane were imaged and counted by using an inverted microscope.

#### BM-MSC osteogenic differentiation assay

When the cells were 60 %–70 % confluent, the complete medium was replaced with osteogenic and induction medium (Cyagen Bioscience Inc.). After incubation for 3–4 weeks, bone nodules were stained with Alizarin Red S.

#### BM-MSC adipocyte differentiation assay

When the cells were 70 %–80 % confluent, the complete medium was replaced with an adipogenic induction medium (Cyagen Bioscience Inc.). After incubation for 2 weeks, lipid droplets were stained by using Oil Red O.

### Extraction and identification of MVs

Cells were starved overnight in serum-free medium supplemented with 0.5 % bovine serum albumin (Sigma-Aldrich, St. Louis, MO, USA) prior to collection of MVs. Cell debris was removed from the supernatant after centrifugation at 2000 × *g* for 20 min. The supernatant was collected and centrifuged at 100,000 × *g* at 4 °C for 1 h by using a high-speed refrigerated centrifuge (CP100WX; Hitachi, Tokyo, Japan). The pellet was resuspended in serum-free RPMI 1640 medium, which was again centrifuged at 100,000 × *g* at 4 °C for 1 h. The final pellet was considered to consist of MVs [17]. The collected MVs were treated with phosphate-buffered saline (PBS) and fluorescein isothiocyanate (FITC) or phycoerythrin (PE)/CY7-labeled anti-CD44, CD29, and alpha 4-integrin antibodies (BioLegend Inc.). Murine IgG labeled with FITC or PE/CY7 (BioLegend Inc.) was used as a negative control. Flow cytometry was used to identify cell surface markers.

### SA-β-Gal staining

A senescence-associated beta-galactosidase (SA-β-Gal) staining kit was used (Beyotime Institute of Biotechnology). P2 BM-MSCs were seeded onto a six-well plate. When cells reached 70 % confluency, the medium was aspirated and the cells were washed twice with PBS. The cells were subsequently fixed with 4 % formaldehyde for 30 min and then stained for 16 h at 37 °C with an SA-β-Gal staining reagent. Positively stained (blue) cells were counted by using an inverted microscope, and the positive rates between the young and elder groups were compared.

### Microarray analysis of miRNAs in BM-MSC-MVs

The technology of miRCURY LNA Array (version 18.0) (Exiqon, Vedbaek, Denmark) was adopted. RNA was extracted and purified from BM-MSC-MVs of both young and elder groups. With an Exiqon miRCURY Hy3/Hy5 Power Labeling Kit, miRNAs were fluorescently labeled and then hybridized in an miRCURY LNA Array Station (version 18.0). A GenePix 4000B microarray reader was used to measure chip fluorescence intensity. Then the fluorescence intensity was converted to raw numeric data by using GenePix pro version 6.0. Triplicates were set up for both young and elder groups. Signals with fluorescence intensities of 30 or above were selected for group analysis. The raw signals were normalized with the median fluorescence intensity of each chip. With the normalized signals, different expression levels of miRNAs between the young and elder groups were calculated. The statistical significance of the differences in miRNA expression between both groups was calculated by using the *t* test. miRNAs with 1.5-fold or above expression difference and *P* values of less than 0.05 were selected and defined as those showing significant differential expression. The microarray data were deposited in the National Center for Biotechnology Information Gene Expression Omnibus (GEO) public repository and are accessible under GEO Series accession number GSE72198.

### Verification of mRNA differential expression in BM-MSC-MVs and sera

Blood from five 3-month-old and five 24-month-old Fisher344 rats was collected from the orbital sinus. After centrifugation at 3000 × *g* for 15 min, 100 μl of serum was collected from the supernatant. Based on methods earlier described, BM-MSCs were extracted and cultured, and the derived MVs were collected from the 10 rats. Total RNAs in sera and MVs were extracted by using an Exiqon miRCURY RNA Isolation Kit. The corresponding cDNAs were synthesized by using SYBR PrimeScript miRNA reverse transcription-polymerase chain reaction (RT-PCR) Kit (Takara, Tokyo, Japan). Lastly, an ABI-Prism 7500 Sequence Detection System (Applied Biosystems, Waltham, MA, USA) and SYBR PrimeScript miRNA RT-PCR Kit (Takara) were used to detect the expression level of specific miRNAs (miR-344a, miR-294, miR-872-3p, miR-133b-3p, and miR-423-3p) and the loading control, miR-191.

### TGF-β1 stimulation and miRNA transfection

HK2 cells (American Type Culture Collection, Manassas, VA, USA) were cultured in 10 % fetal bovine serum (FBS) Dulbecco’s modified Eagle’s medium/F12 (DMEM/F12) medium (Corning). When the cells reached 50 % confluency, serum-free DMEM/F12 medium was used to synchronize the cells for 18 h. The HK2 cells were incubated with recombinant human TGF-β1 (PeproTech Inc., Rocky Hill, NJ, USA) at a concentration of 8 ng/ml for 48 h to induce fibrosis with young MV (10^5^ young MSCs released overnight) or old MV (10^5^ old MSCs released overnight) treatment. For the miRNA transfection groups, miR-344a, miR-294, miR-872-3p, miR-133b-3p, and miRNA mimic control (GeneCopoeia Inc., Rockville, MD, USA) were transfected into the synchronized HK2 cells in accordance with the instructions of the jetPRIME® transfection reagent (Polyplus Transfection Inc., USA). Approximately 24 h after transfection, the medium was replaced with 10 % FBS DMEM/F12 medium (Corning, Shanghai, China) containing 8 ng/ml of TGF-β1 (PeproTech Inc.) and then incubated for 48 h.

### Western blot analysis

The HK2 cells were lysed with RIPA lysis buffer, and then 40 μg of the total protein extract was loaded into each well and separated by using 8 % polyacrylamide (SDS-PAGE) protein gel electrophoresis. The proteins were transferred onto nitrocellulose membranes. The membranes were blocked in 1× casein (Sigma-Aldrich) for 1 h, and the membranes were incubated with the following primary antibodies at 4 °C overnight:

i. Rabbit monoclonal anti-E-cadherin (1:800 dilution; Biogot Biotechnology Co. Ltd., Nanjing, China)
ii. Rabbit monoclonal anti-alpha-smooth muscle actin (anti-α-SMA) (1:500 dilution; Abcam, Cambridge, MA, USA)
iii. Anti-β-actin (1:5,000 dilution; Beyotime Institute of Biotechnology).

After excess primary antibodies were washed off, the membranes were incubated with secondary antibodies (Beyotime Institute of Biotechnology) diluted in 1:1000 for 2 h at room temperature. The target band was detected chemiluminescently by using an enhanced chemiluminescence (ECL) Western Blotting kit (Applygen Technologies Inc., Beijing, China). While β-actin was used as internal control, the relative expression levels of E-cadherin and α-SMA were calculated in each experimental group.

### Immunofluorescence

HK2 cell suspensions were seeded onto autoclaved glass coverslips placed in six-well plates (10^5^ cells per well). After overnight incubation at 37 °C in 5 % CO_2_, cells had adhered to the coverslips. After synchronization and transfection based on previous methods, 10 % FBS DMEM/F12 medium (Corning) with or without 8 ng/ml TGF-β1 (PeproTech Inc.) was used, and the cells were further incubated for 48 h. After fixing in 4 % paraformaldehyde (Beyotime Institute of Biotechnology) at room temperature for 15 min, the cells were permeabilized in 0.2 % Triton X-100 (Sigma-Aldrich) for 5 min and then blocked in 1× casein (Sigma-Aldrich) at room temperature for 30 min. Rabbit monoclonal anti-E-cadherin (1:100, Biogot Biotechnology Co. Ltd.) and anti-α-SMA (1:50; Abcam) primary antibodies were diluted and added to the coverslips and then incubated overnight at 4 °C. After washing with PBS, fluorescent anti-rabbit conjugated with CY3 or FITC secondary antibodies (1:400, Jackson ImmunoResearch Laboratories, Bar Harbor, ME, USA) were added and incubated for 2 h at room temperature. After washing with PBS, the coverslips were removed from the six-well plates and placed on a glass slide, and mounting medium containing 4ʹ,6-diamidino-2-phenylindole (DAPI) (ZSGB-BIO, Beijing, China) was added. Random fields were chosen under a fluorescence microscope (Olympus America Inc., Center Valley, PA, USA), and the expressions of E-cadherin and α-SMA in HK2 cells were observed.

### Statistical analysis

Results were expressed as the mean ± standard deviation by using SPSS 17.0 software (IBM Corporation, Armonk, NY, USA). Differences between experimental groups were analyzed by using one-way analysis of variance. *P* values of less than 0.05 were considered statistically significant.

## Results

### Identification and senescence staining of BM-MSCs

**Fig. 1:**
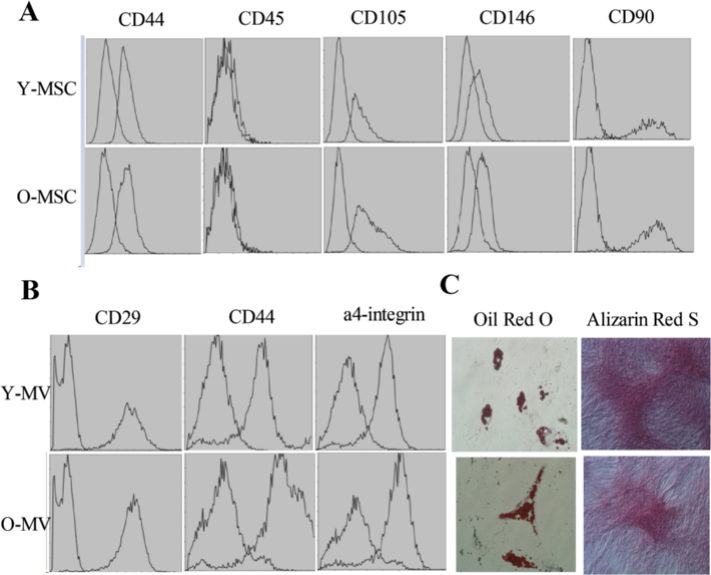
Characterization of young and old MSCs as well as young and old MVs. **a** BM-MSCs from young and old Fisher344 rats were cultured to passage 3 and labeled with a FITC-coupled antibodies against CD44 and CD146 and a PE/Cy7-coupled antibody against CD45 and CD105 or immunoglobulin isotype control IgGs and analyzed by using FACS. **b** MVs from young and old MSCs were collected and labeled with FITC-coupled antibodies against CD44, a4-integrin, and PE/Cy7-coupled antibody against CD29 or immunoglobulin isotype control IgGs and analyzed by using FACS. **c** Representative photomicrographs show lipid inclusions with Oil Red O (*left*) and mineralization with alizarin red (*right*) in MSCs from young (*top*) and old rats (*bottom*). *FACS* fluorescence-activated cell sorting, *FITC* fluorescein isothiocyanate, *MSC* mesenchymal stem cell, *MV* microvesicle, *PE* phycoerythrin

Figure S1 in Additional file 1 shows that fewer BM-MSCs were extracted from an equal amount of bone marrow in older, 24-month-old rats compared with younger, 3-month-old rats. Moreover, under the same incubation conditions, we observed fewer adherent BM-MSCs in the older group than in the younger rats. In addition, the growth rate of BM-MSCs derived from young rats was significantly faster than those derived from older rats. With each additional passage of primary BM-MSCs derived from older rats, we observed slower and irregular cell growth, with wider intercellular spacing in BM-MSCs (Additional file 1). By using SA-β-Gal staining that detects senescent cells, we observed a higher positive staining rate in BM-MSCs from older rats than in BM-MSCs from younger rats (Additional file 2). According to the flow cytometry data, the expression of BM-MSC surface markers, including CD44, CD105, and CD146, was positive in both young and older rats, whereas the expression of CD45 was negative in both groups (Fig. 1a). Adhesion molecules, including CD44, CD29, and α4-integrin, which are expressed on the plasma membrane of BM-MSCs, were detected on the surface of BM-MSC-MVs from both young and elder rats (Fig. 1b). BM-MSCs from both young and elder rats had the capacity to differentiate into adipocytes and osteoblasts (Fig. 1c).

### Aged BM-MSCs show decreased proliferation and migration

BM-MSCs of older rats showed significantly slower growth rate *in vitro* than that of younger rats (Additional file 3a). In the transwell migration assay, compared with BM-MSCs derived from younger rats, the migration capacity of BM-MSCs from older rats also remarkably decreased, and this was likely due to aging (Additional file 3b).

### Change in miRNA expression profiles in BM-MSC-MVs caused by aging

**Fig. 2:**
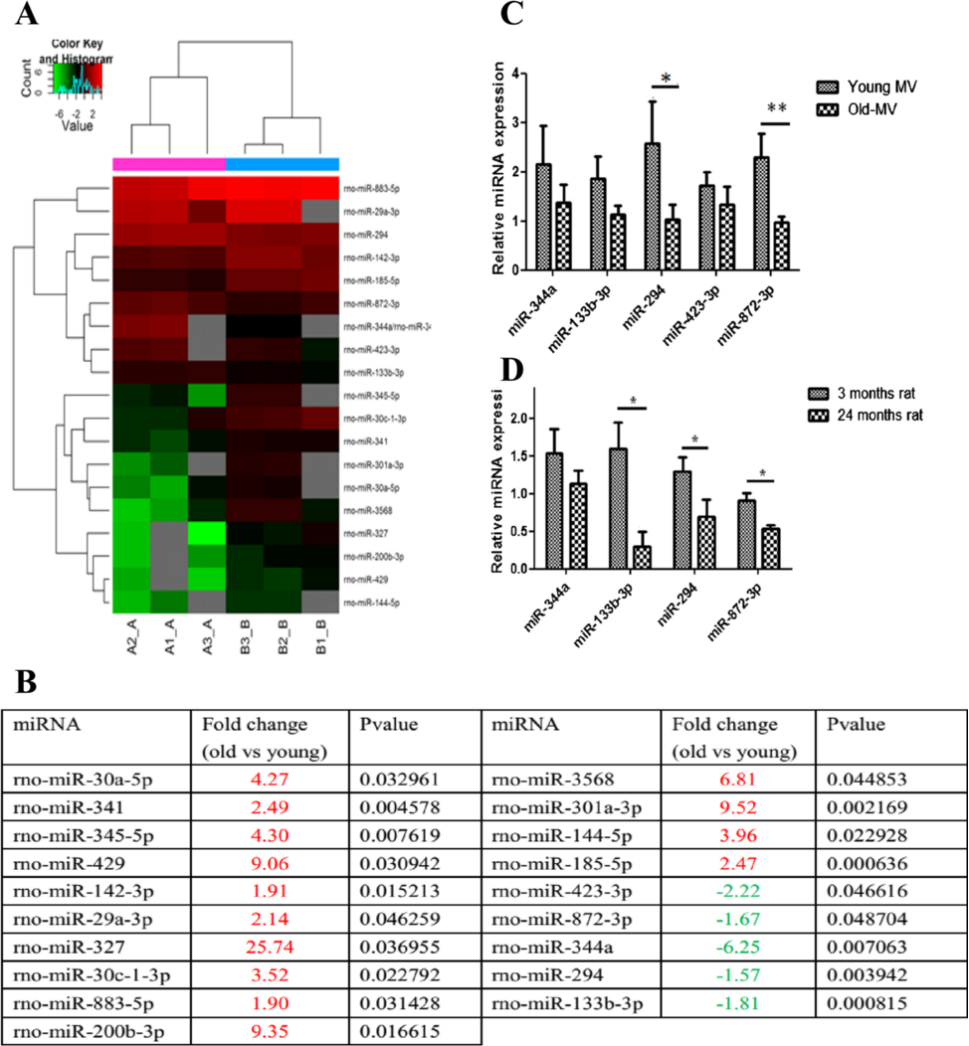
Age-dependent changes in miRNA profiles of young and old MVs. **a** Expression of miRNAs in young and old MVs and mapping of hierarchic clustering of miRNAs and heatmap display of miRNA profiles. **b** Significant fold changes in miRNAs (fold change > 1.5) in old MVs versus young MVs. Upregulated miRNAs were marked in *red*, and downregulated miRNAs are indicated in *green*. **c** RT-PCR analysis was used to validate the downregulated miRNAs in young and old MVs. **d** RT-PCR analysis was employed to validate the downregulated miRNAs in serum from young and old rats (*P < 0.05; **P < 0.01; n = 5). *miRNA* microRNA, *MV* microvesicle, *RT-PCR* reverse transcription-polymerase chain reaction

Compared with the younger group, 117 miRNAs from BM-MSC-MVs derived from older rats were differentially expressed, and 19 of them showed statistically significant changes (*P* < 0.05): the expression levels of 14 miRNAs were upregulated and 5 were downregulated (Fig. 2a, b). For significantly downregulated miRNAs on the microarray, the expression levels in MVs were verified by RT-PCR, and the results from both assays were in agreement. miR-344a, miR-133b-3p, miR-294, miR-423-3p, and miR-872-3p in BM-MSC-MVs from aged rats were downregulated, and the expression of miR-294 and miR-872-3p showed a significant decline (*P* < 0.05) (Fig. 2c).

### Expression of miRNAs in blood

Compared with younger rats, the serum levels of miR-344a, miR-133b-3p, miR-294, and miR-872-3p in aged rats decreased, and those of miR-133b-3p, miR-294, and miR-872-3p showed a significant decrease (*P* < 0.05) (Fig. 2d).

### Inhibition of TGF-β1-mediated EMT by BM-MSC-MV is weakened in older rats

**Fig. 3:**
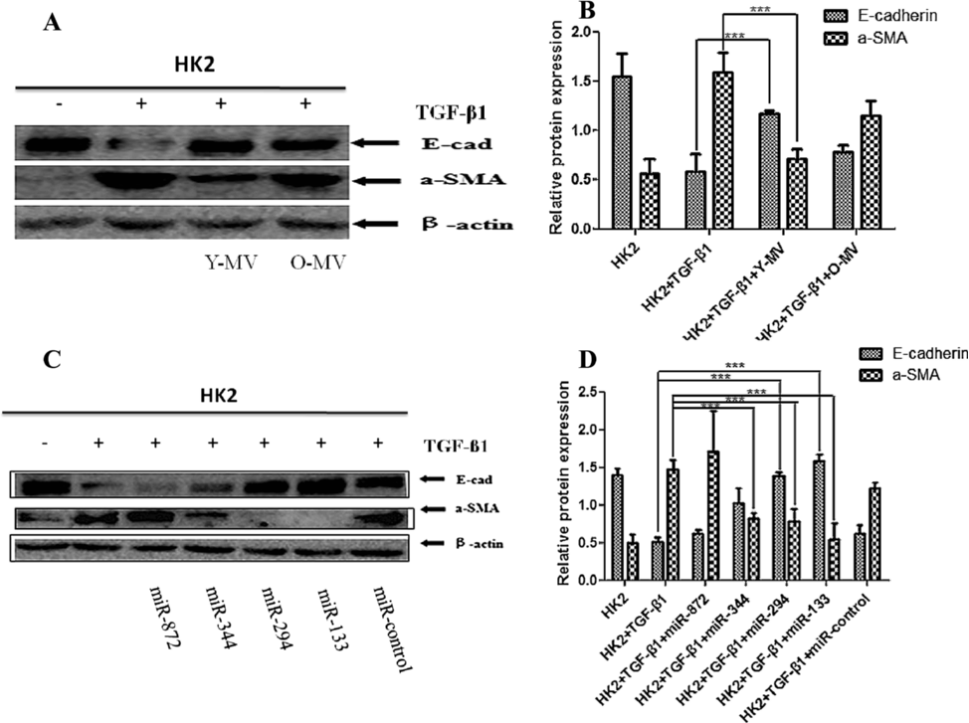
Effect of MVs and miRNAs on the inhibitory effect of TGF-β1 on the EMT in HK2 cells. **a, c** Western blot analysis of E-cadherin and α-SMA expression in HK2 cells under TGF-β1 stimulation co-cultured with Y-MV /O-MV or pre-transfection with miR-872, miR-344, miR-294, miR-133b -3p, and miR-control mimic. β-actin was used as internal control. **b, d** Quantification of E-cadherin and α-SMA protein levels in HK2 cells from each experimental group. **P < 0.05; n =3. *α-SMA* alpha-smooth muscle actin, *EMT* epithelial-mesenchymal transition, *MV* microvesicle, *TGF-β1* transforming growth factor-beta 1

**Fig. 4:**
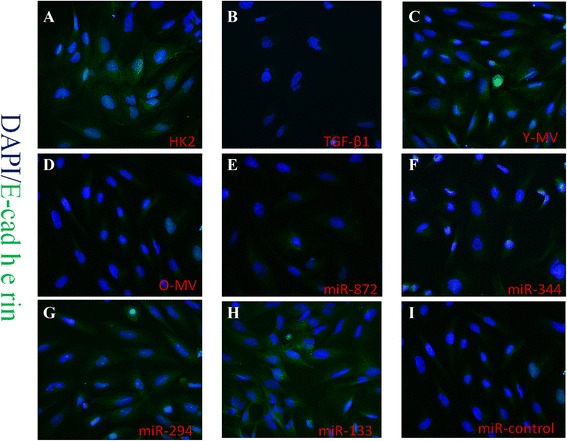
Immunocytochemical analysis of E-cadherin staining: Representative images of anti-E-cadherin staining in HK2 cells from each experimental group. The *green* staining shows the expression of E-cadherin in normal HK2 cells that were (**a**) treated with TGF-β1 (**b**) or co-cultured with Y-MV (**c**) or Old-MV (**d**). It also indicated the expression of E-cadherin in HK2 cells treated with TGF-β1 but pre-transfected with miR-872, miR-344, miR-294, miR-133b-3p, and miR-control mimic (**e-i**). *TGF-β1* transforming growth factor-beta 1

**Fig. 5:**
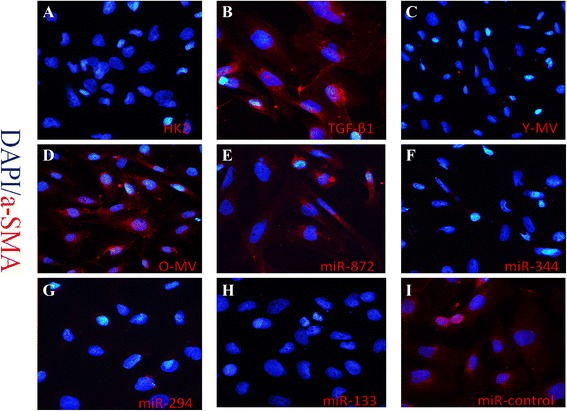
Immunocytochemical staining of α-SMA in HK2 cells from each experimental group. The *red* staining shows the expression of α-SMA in normal HK2 cells that were (**a**) treated with TGF-β1 (**b**) or co-cultured with Y-MV (**c**) or Old-MV (**d**). It also indicated the expression of α-SMA in HK2 cells treated with TGF-β1 but pre-transfected with miR-872, miR-344, miR-294, miR-133b-3p, and miR-control mimic (**e–i**). *α-SMA* alpha-smooth muscle actin, *TGF-β1* transforming growth factor-beta 1

BM-MSC-MVs derived from younger rats showed significant inhibitory effects on TGF-β1-mediated EMT in HK2 cells. The addition of BM-MSC-MVs from younger rats to TGF-β1 resulted in the stimulation of HK2 cells to reverse the downregulated E-cadherin expression and upregulated α-SMA expression (*P* < 0.05 for both). On the other hand, BM-MSC-MVs derived from older rats did not show such effects (Fig. 3a, b). These observations of EMT marker expression in HK2 cells subjected to various treatments were further confirmed by immunofluorescence staining (Figs. 4a–d and 5a–d).

### Effects of miRNA downregulated in elder rat BM-MSC-MVs on TGF-β1-mediated EMT

HK2 cells were initially transfected with miR-344a, miR-294, miR-872 -3p, miR-133b-3p, and miRNA mimic control and then incubated in a medium containing 8 ng/ml TGF-β1 for 48 h. Compared with non-transfected HK2 cells, the expression level of α-SMA significantly decreased in HK2 cells transfected with miR-344a, miR-294, and miR-133b-3p (*P* < 0.05), and the expression level of E-cadherin significantly increased in HK2 cells transfected with miR-294 and miR-133b-3p (*P* < 0.05). Meanwhile, HK2 cells transfected with miR-872-3p exhibited no significant changes (Fig. 3c, d). We also validated these results by immunofluorescence staining, which showed that HK2 cells without TGF-β1 stimulation displayed high expression of E-cadherin and low expression of α-SMA. After TGF-β1 stimulation, HK2 cells presented lower expression of E-cadherin and higher expression of α-SMA. Moreover, miR-344a, miR-294, and miR-133b-3p weakened the upregulation of α-SMA and inhibited the downregulation of E-cadherin in HK2 cells induced by TGF-β1 (Figs. 4e-i and 5e-i).

## Discussion

Renal interstitial fibrosis is a characteristic pathology of aging kidneys and the basis of CKD progression. EMT of renal tubules is the leading cause of renal interstitial fibrosis. Several studies have shown that TGF-β1 enhances EMT through a variety of mechanisms [21–23]. In addition, various studies have demonstrated that BM-MSCs participate in renal tissue repair. Morigi et al. [24] showed that BM-MSCs can improve kidney functions, and BM-MSCs are the primary cell population involved in renal tubular and functional repair. Other recent investigations have also shown that BM-MSCs can reduce renal damage through paracrine activity [25–27].

MVs are small circular diaphragms (diameter of 100 nm to 1 μm) shed from the cell surface or released from cellular compartments. MVs contain proteins, enzymes, mRNA, and miRNA and may participate in tissue damage repair through paracrine activity [15, 17, 27]. Camussi et al. [15] revealed that both BM-MSCs and BM-MSC-MVs promote morphological restoration and renal function maintenance in animals with renal ischemia-reperfusion or unilateral nephrectomy. MVs secreted by BM-MSCs can enter the damaged kidney cells and play a role in kidney repair. Cantaluppi et al. [28] also observed that MVs in rats possessed a repair function for renal ischemia-reperfusion injury. However, pre-treatment with an efficient miRNA blocker resulted in a reduction in repair function, suggesting that MVs can transfer miRNAs to repair damaged kidneys.

In eukaryotes, miRNAs play an extremely important role in organ development and maturation as well as the pathogenesis and progression of disease [29]. Previous studies have shown that in fibrotic renal tissues the expressions of both miR-200 family and E-cadherin are significantly downregulated and that exogenous miR-200 could reduce renal fibrosis [30, 31]. Pre-transfection of the miR-200 family can reverse TGF-β1-mediated EMT in tubular epithelial cells [30, 32, 33]. Through the regulation of Tgfbr2 and Gsk3-beta, miR-294/302 can inhibit TGF-beta and GSK3 pathways. On the other hand, miR-294/302 can indirectly inhibit Tgfbr2 as well as EMT of embryonic stem cells [34]. Liu et al. discovered that, as a downstream gene of fibrotic TGF-β/Smad3 pathway, miR-133 can negatively regulate TGF-β/Smad3 [35]. Along with physiological aging, BM-MSCs also age, and the cellular molecules these secrete significantly decrease or even disrupt secretion. Expression profiles of miRNAs can also significantly change with age, and its functions against diseases can decrease or become ineffective [36, 37].

In the present study, we found that the number of BM-MSCs derived from 24-month-old rats was significantly lower, displayed a senescent phenotype, and had a markedly slower *in vitro* proliferation rate compared with that observed in 3-month-old rats. Furthermore, these aged BM-MSCs showed irregular growth with increased cellular spacing, and their migration ability was significantly weaker. We also observed significant differences in miRNA expression of BM-MSC-MVs between younger and older rats. Compared with younger rats, five miRNAs in BM-MSC-MVs—miR-344a, miR-133b-3p, miR-294, miR-423-3p, and miR-872-3p—were significantly reduced in older rats. Additionally, compared with MVs from younger rats, the inhibitory effects of MVs from older rats on TGF-β1-induced EMTs were significantly reduced. We also observed that miR-133b-3p and miR-294, which were downregulated in older rats, imparted significant inhibitory effects on TGF-β1-induced EMT. Our data therefore suggested that the downregulation of miR-133b-3p and miR-294 in older rats may be an important cause of suppression of TGF-β1-induced EMT in aged rats.

In recent years, studies have shown that changes in body environment affect aging of organs. Through the establishment of Siamese animal models such as young and older rats, we observed that learning and memorization were enhanced in older rats but that these were weakened in younger rats. With injection of serum from older rats, younger rats presented brain aging phenotypes. Conversely, injection of serum from younger rats resulted in the alleviation of the brain aging phenotype in older rats [38]. In the young-elderly rat model, cardiac hypertrophy of aged hearts was significantly less than that of the elder-elder rat model, and cardiac function significantly improved [39]. In addition, in the young-elderly rat model, aged muscles, liver, neural stem/progenitor cells, and ovarian follicles were activated, and the regeneration capacity of tissues and organs was enhanced [40–42]. The miRNAs contained in BM-MSC-MVs can be released into blood circulation, where they are converted into circulating miRNAs that possess the ability to change the internal environment, and ultimately impart systemic effects. Our current experimental results showed that miRNAs that were downregulated in MVs from older rats also exhibited a comparatively low level in serum, suggesting that by secreting MVs and changing miRNA expression, BM-MSCs changed the body environment during the aging process. In older rats, the level of secreted miR-133b-3p and miR-294 decreased. Their levels in serum were also low and this may be an important factor in causing renal EMT and renal aging.

In the present study, we confirmed that, in older rats, the inhibitory effects of BM-MSC-MVs on TGF-β1-induced EMT had weakened, and this might be related to the decrease in the levels of miR-133b-3p and miR-294. However, the target genes, regulatory mechanisms, and signaling pathways of miR-133b-3p and miR-294 currently remain unclear and should be investigated in future studies.

## Conclusions

The present study observed that the inhibitory effect of BM-MSC-MVs on TGF-β1-mediated HK2 cell EMT was weaker in older rats than in younger rats. Moreover, miR-133b-3p and miR-294, which were downregulated in BM-MSC-MVs and serum of older rats, could remarkably inhibit TGF-β1-mediated EMT in HK2 cells. These findings suggest that the downregulated miR-133b-3p and miR-294 in older rats play important roles in causing renal EMT and renal aging.

## Additional files

Additional file 1:
**Cell morphology and population size in young and old MSCs.** Representative phase-contrast micrographs of cultured BM-MSCs derived from young (*bottom*) and old rats (*top*) of the P_0_, P_1_, and P_2_ generations. *BM-MSC* bone marrow mesenchymal stem cell, *MSC* mesenchymal stem cell. (DOC 2450 kb) Additional file 2:
**SA-β-gal expression in young and old MSCs.** **a** SA-β-gal staining (×100). Compared with the Y-MSC group, the number of SA-β-gal-positive cells in the O-MSC group clearly increased. **b** Quantification of SA-β-gal-positive cells. The number of SA-β-gal-positive cells was determined by screening 500 random cells under a phase-contrast microscope. The number of SA-β-gal-positive cells in the O-MSC group was significantly higher than that in the Y-MSC group (***P* < 0.01; n = 5). *MSC* mesenchymal stem cell, *SA-β-gal* senescence-associated beta-galactosidase. (DOC 2015 kb) Additional file 3:
**The proliferation and migration ability of young and old MSCs.** **a** CCK-8 assay shows that the proliferation of the MSCs was not significantly different between the young and old MSCs on days 1 and 2. The absorbance value in the old MSCs was significantly lower than that in the young MSCs on days 3 and 4. **b** The number of transferred MSCs in the young group (*top*) was significantly higher than that in the old group (*bottom*). (**P* < 0.05; n = 5). *MSC* mesenchymal stem cell. (DOC 2433 kb)

## Abbreviations

α-SMA: Alpha-smooth muscle actin
BM-MSC: Bone marrow mesenchymal stem cell
BM-MSC-MV: Bone marrow mesenchymal stem cell-derived microvesicle
CKD: Chronic kidney disease
DMEM/F12: Dulbecco’s modified Eagle’s medium/F12
EMT: Epithelial-mesenchymal transition
FBS: Fetal bovine serum
FITC: Fluorescein isothiocyanate
GEO: Gene Expression Omnibus
miRNA: MicroRNA
MV: Microvesicle
PBS: Phosphate-buffered saline
PE: Phycoerythrin
RT-PCR: Reverse transcription-polymerase chain reaction
SA-β-Gal: Senescence-associated beta-galactosidase
TGF-β1: Transforming growth factor-beta 1

## References

[1] Zhang QL, Rothenbacher D (2008). Prevalence of chronic kidney disease in population-based studies: systematic review. BMC Public Health..

[2] Zhang L, Wang F, Wang L, Wang W, Liu B, Liu J (2012). Prevalence of chronic kidney disease in China: a cross-sectional survey. Lance..

[3] Coresh J, Selvin E, Stevens LA, Manzi J, Kusek JW, Eggers P (2007). Prevalence of chronic kidney disease in the United States. JAMA..

[4] Gewin L, Zent R (2012). How does TGF-β mediate tubulointerstitial fibrosis?. Semin Nephrol..

[5] Gimble JM, Guilak F, Bunnell BA (2010). Clinical and preclinical translation of cellbased therapies using adipose tissue-derived cells. Stem Cell Res Ther..

[6] Izadpanah R, Joswig T, Tsien F, Dufour J, Kirijan JC, Bunnell BA (2005). Characterization of multipotent mesenchymal stem cells from the bone marrow of rhesus macaques. Stem Cells Dev..

[7] Prockop DJ (2009). Repair of tissues by adult stem/progenitor cells (MSCs): controversies, myths, and changing paradigms. Mol Ther..

[8] Tokalov SV, Grüner S, Schindler S, Wolf G, Baumann M, Abolmaali N (2007). Agerelated changes in the frequency of mesenchymal stem cells in the bone marrow of rats. Stem Cells Dev..

[9] Zhou S, Greenberger JS, Epperly MW, Goff JP, Adler C, Leboff MS (2008). Age-related intrinsic changes in human bone-marrowderived mesenchymal stem cells and their differentiation to osteoblasts. Aging Cell..

[10] Yu JM, Wu X, Gimble JM, Guan X, Freitas MA, Bunnell BA (2011). Age-related changes in mesenchymal stem cells derived from rhesus macaque bone marrow. Aging Cell..

[11] Stolzing A, Jones E, McGonagle D, Scutt A (2008). Age-related changes in human bone marrow-derived mesenchymal stem cells: consequences for cell therapies. Mech Ageing Dev..

[12] Van Zant G, Liang Y (2003). The role of stem cells in aging. Exp Hematol..

[13] Carrington JL (2005). Aging bone and cartilage: cross-cutting issues. BiochemBiophys Res Commun..

[14] Rao MS, Mattson MP (2001). Stem cells and aging: expanding the possibilities. Mech Ageing Dev..

[15] Gatti S, Bruno S, Deregibus MC, Sordi A, Cantaluppi V, Tetta C (2011). Microvesicles derived from human adult mesenchymal stem cells protect against ischaemia-reperfusion-induced acute and chronic kidney injury. Nephrol Dial Transplant..

[16] He J, Wang Y, Sun S, Yu M, Wang C, Pei X (2012). Bone marrow stem cells-derived microvesicles protect against renal injury in the mouse remnant kidney model. Nephrology..

[17] Bruno S, Grange C, Deregibus MC, Calogero RA, Saviozzi S, Collino F (2009). Mesenchymal stem cell-derived microvesicles protect against acute tubular injury. J Am Soc Nephrol..

[18] Hackl M, Brunner S, Fortschegger K, Schreiner C, Micutkova L, Mück C (2010). miR-17, miR-19b, miR-20a, and miR-106a are down-regulated in human aging. Aging Cell.

[19] Liang R, Bates DJ, Wang E (2009). Epigenetic control of microRNA expression and aging. Curr Genomics..

[20] Noren Hooten N, Abdelmohsen K, Gorospe M, Ejiogu N, Zonderman AB, Evans MK (2010). microRNA expression patterns reveal differential expression of target genes with age. PLoS One.

[21] Bottinger B (2002). TGF-beta signaling in renal disease. J Am Soc Nephrol..

[22] Samarakoon R, Overstreet JM, Higgins SP, Higgins PJ (2012). TGF-beta1 –> SMAD/p53/USF2 –> PAI-1 transcriptional axis in ureteral obstruction-induced renal fibrosis. Cell Tissue Res..

[23] López-Hernández FJ, López-Novoa JM (2012). Role of TGF-beta in chronic kidney disease: an integration of tubular, glomerular and vascular effects. Cell Tissue Res..

[24] Morigi M, Imberti B, Zoja C, Corna D, Tomasoni S, Abbate M (2004). Mesenchymal stem cells are renotropic, helping to repair the kidney and improve function in acute renal failure. J Am Soc Nephrol..

[25] Ratajczak J, Miekus K, Kucia M, Zhang J, Reca R, Dvorak P (2006). Embryonic stem cells-derived microvesicles reprogram hematopoietic progenitors: Evidence for horizontal transfer of mRNA and protein delivery. Leukemia..

[26] Tetta C, Bruno S, Fonsato V, Deregibus MC, Camussi G (2011). The role of microvesicles in tissue repair. Organogenesis..

[27] Collino F, Deregibus MC, Bruno S, Sterpone L, Aghemo G, Viltono L (2010). Microvesicles derived from adult human bone marrow and tissue specific mesenchymal stem cells shuttle selected pattern of miRNAs. PLoS One..

[28] Cantaluppi V, Gatti S, Medica D, Figliolini F, Bruno S, Deregibus MC (2012). Microvesicles derived from endothelial progenitor cells protect the kidney from ischemia–reperfusion injury by microRNA-dependent reprogramming of resident renal cells. Kidney Int..

[29] Zhao Y, Srivastava D (2007). A development view of microRNA function. Trends in Biochem Sci..

[30] Mongrco PS, Rustgi AK (2010). The role of the miR-200 family in epithelial—mesenchymal transition. Cancer Bid Ther..

[31] Gregory PA, Bert AG, Paterson EL, Barry SC, Tsykin A, Farshid G (2008). The miR-200 family and miR-205 regulate epithelial to mesenchymal transition by targeting ZEB1 and SIP1. Nat Cell Biol..

[32] Park SM, Gaur AB, Lengyel E, Peter ME (2008). The miR-200 family determines the epithelial phenotype of cancer cells by targeting the E-cadherin repressors ZEB1 and ZEB2. Genes Dev..

[33] Gregory PA, Bracken CP, Bert AG (2008). Micro-RNAs as regulators of epithelial-mesenchymal transition. Cell Cycle..

[34] Guo WT, Wang XW, Yan YL, Li YP, Yin X, Zhang Q (2014). Suppression of epithelial-mesenchymal transition and apoptotic pathways by miR-294/302 family synergistically blocks let-7-induced silencing of self-renewal in embryonic stem cells. Cell Death Differ..

[35] Duan LJ, Qi J, Kong XJ, Huang T, Qian XQ, Xu D (2015). MiR-133 modulates TGF-β1-induced bladder smooth muscle cell hypertrophic and fibrotic response: implication for a role of microRNA in bladder wall remodeling caused by bladder outlet obstruction. Cell Signal..

[36] Asumda FZ, Chase PB (2011). Age-related changes in rat bone-marrow mesenchymal stem cell plasticity. BMC Cell Biol..

[37] Pandey AC, Semon JA, Kaushal D, O'Sullivan RP, Glowacki J, Gimble JM (2011). MicroRNA profiling reveals age-dependent differential expression of nuclear factor κB and mitogen-activated protein kinase in adipose and bone marrow-derived human mesenchymal stem cells. Stem Cell Res Ther..

[38] Villeda SA, Luo J, Mosher KI, Zou B, Britschgi M, Bieri G (2011). The ageing systemic milieu negatively regulates neurogenesis and cognitive function. Nature..

[39] Loffredo FS, Steinhauser ML, Jay SM, Gannon J, Pancoast JR, Yalamanchi P, Sinha M (2013). Growth differentiation factor 11 is a circulating factor that reverses age-related cardiac hypertrophy. Cell..

[40] Ruckh JM, Zhao JW, Shadrach JL, van Wijngaarden P, Rao TN, Wagers AJ (2012). Rejuvenation of regeneration in the aging central nervous system. Cell Stem Cell..

[41] Conboy IM, Conboy MJ, Wagers AJ, Girma ER, Weissman IL, Rando TA (2005). Rejuvenation of aged progenitor cells by exposure to a young systemic environment. Nature..

[42] Niikura Y, Niikura T, Wang N, Satirapod C, Tilly JL (2010). Systemic signals in aged males exert potent rejuvenating effects on the ovarian follicle reserve in mammalian females. Aging (Albany NY)..

