# Supplementary material for: Differentially expressed microRNAs in bone marrow mesenchymal stem cell-derived microvesicles in young and older rats and their effect on tumor growth factor-β1-mediated epithelial-mesenchymal transition in HK2 cells
Source: Stem Cell Res Ther. 2015 Sep 28;6:185. doi: 10.1186/s13287-015-0179-x (PMC4587922; doi:10.1186/s13287-015-0179-x)
Supplement: Additional file 1: — Cell morphology and population size in young and old MSCs. Representative phase-contrast micrographs of cultured BM-MSCs derived from young (bottom) and old rats (top) of the P0, P1, and P2 generations. BM-MSC bone marrow mesenchymal stem cell, MSC mesenchymal stem cell. (DOC 2450 kb) [file 13287_2015_179_MOESM1_ESM.doc]

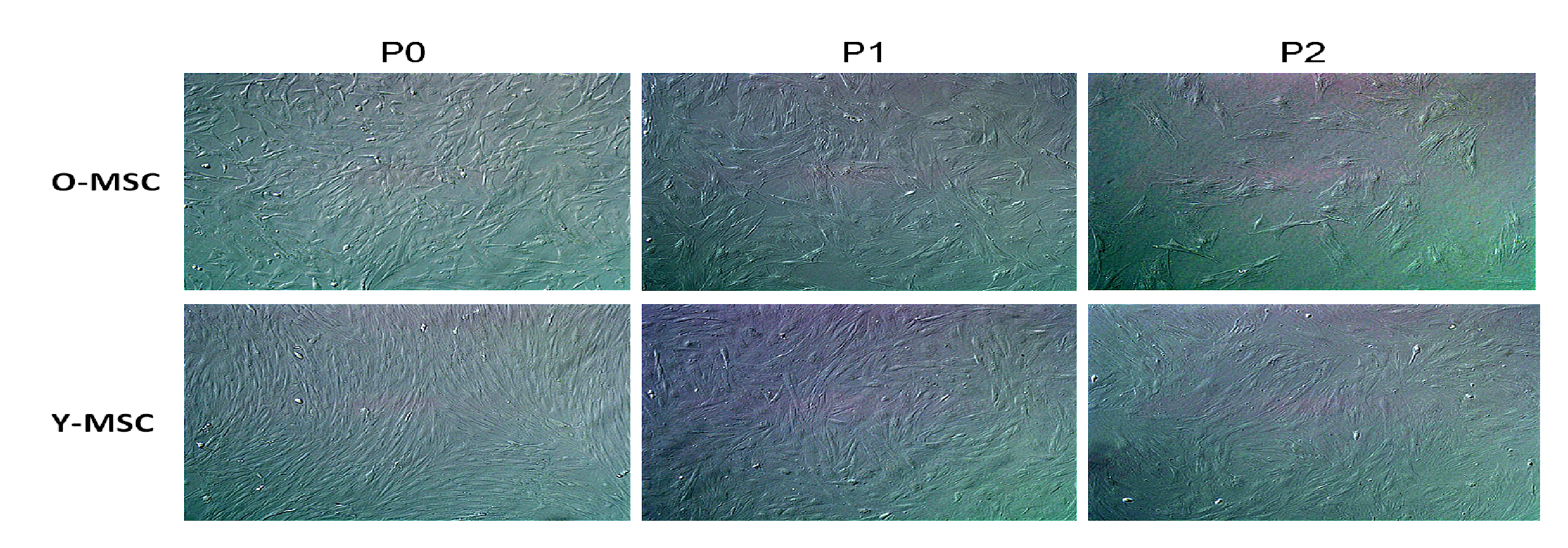


**Additional file 1:** **Cell morphology and population size in young and old MSCs.** Representative phase-contrast micrographs of cultured BM-MSCs derived young (bottom) and old rat(top) at P_0_,P_1_ and P_2_ generation.
