# Supplementary material for: Differentially expressed microRNAs in bone marrow mesenchymal stem cell-derived microvesicles in young and older rats and their effect on tumor growth factor-β1-mediated epithelial-mesenchymal transition in HK2 cells
Source: Stem Cell Res Ther. 2015 Sep 28;6:185. doi: 10.1186/s13287-015-0179-x (PMC4587922; doi:10.1186/s13287-015-0179-x)
Supplement: Additional file 2: — SA-β-gal expression in young and old MSCs. a SA-β-gal staining (×100). Compared with the Y-MSC group, the number of SA-β-gal-positive cells in the O-MSC group clearly increased. b Quantification of SA-β-gal-positive cells. The number of SA-β-gal-positive cells was determined by screening 500 random cells under a phase-contrast microscope. The number of SA-β-gal-positive cells in the O-MSC group was significantly higher than that in the Y-MSC group (**P < 0.01; n = 5). MSC mesenchymal stem cell, SA-β-gal senescence-associated beta-galactosidase. (DOC 2015 kb) [file 13287_2015_179_MOESM2_ESM.doc]

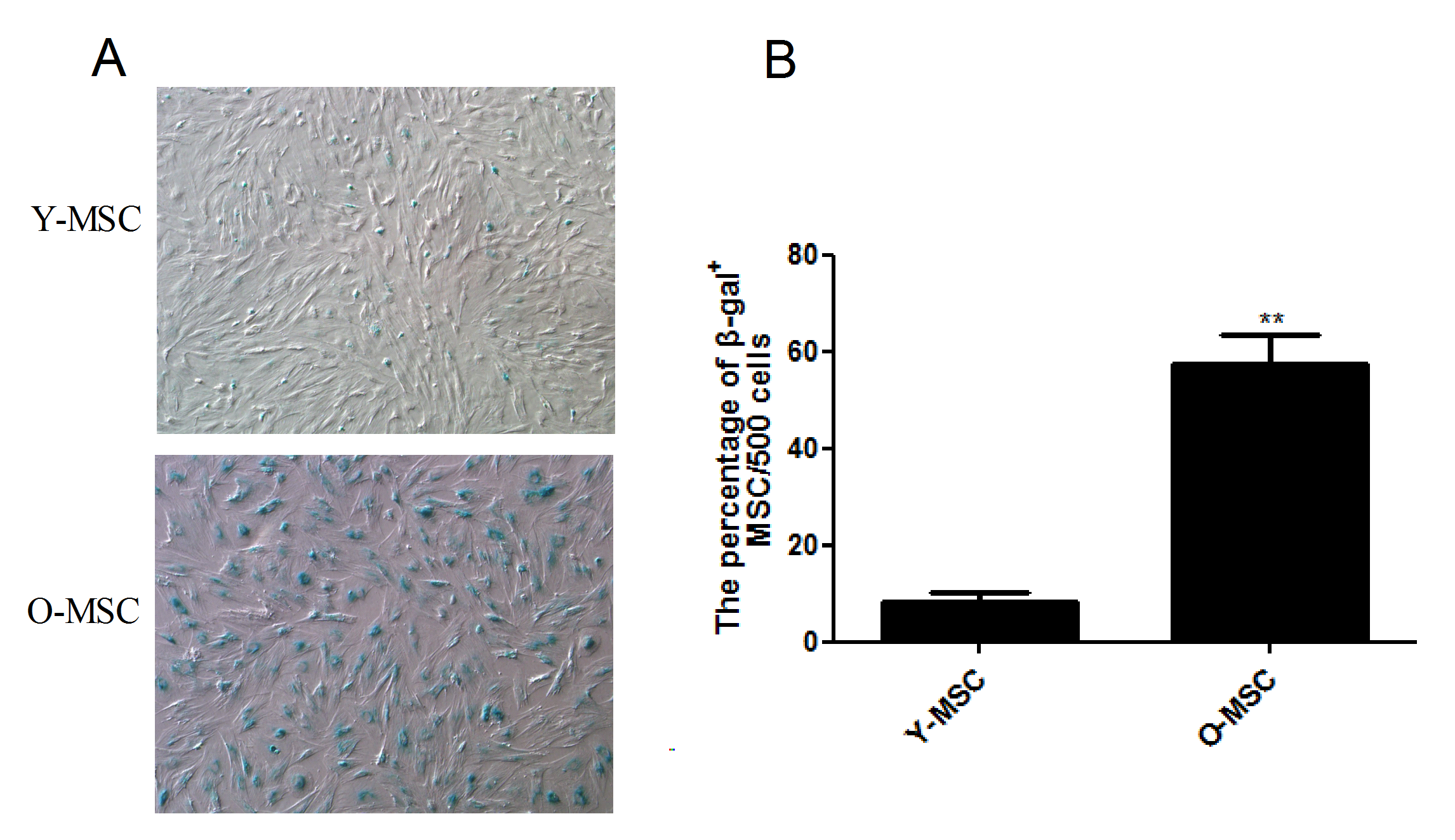


**Additional file 2 SA-b-gal expression in young and old MSCs. (A)** SA-b-gal staining.( ×100)Compared to the Y-MSC group, the number of SA-b-gal-positive cells in the O-MSC group clearly increased. **(B)** Quantification of SA-b-gal-positive cells. The number of SA-b-gal–positive cells was determined by screening 500 random cells under a phase-contrast microscope. The number of SA-b-gal-positive cells in the O-MSC group was significantly higher than that in the Y-MSC group (**P<0.01; n = 5).
