# Supplementary material for: Differentially expressed microRNAs in bone marrow mesenchymal stem cell-derived microvesicles in young and older rats and their effect on tumor growth factor-β1-mediated epithelial-mesenchymal transition in HK2 cells
Source: Stem Cell Res Ther. 2015 Sep 28;6:185. doi: 10.1186/s13287-015-0179-x (PMC4587922; doi:10.1186/s13287-015-0179-x)
Supplement: Additional file 3: — The proliferation and migration ability of young and old MSCs. a CCK-8 assay shows that the proliferation of the MSCs was not significantly different between the young and old MSCs on days 1 and 2. The absorbance value in the old MSCs was significantly lower than that in the young MSCs on days 3 and 4. b The number of transferred MSCs in the young group (top) was significantly higher than that in the old group (bottom). (*P < 0.05; n = 5). MSC mesenchymal stem cell. (DOC 2433 kb) [file 13287_2015_179_MOESM3_ESM.doc]

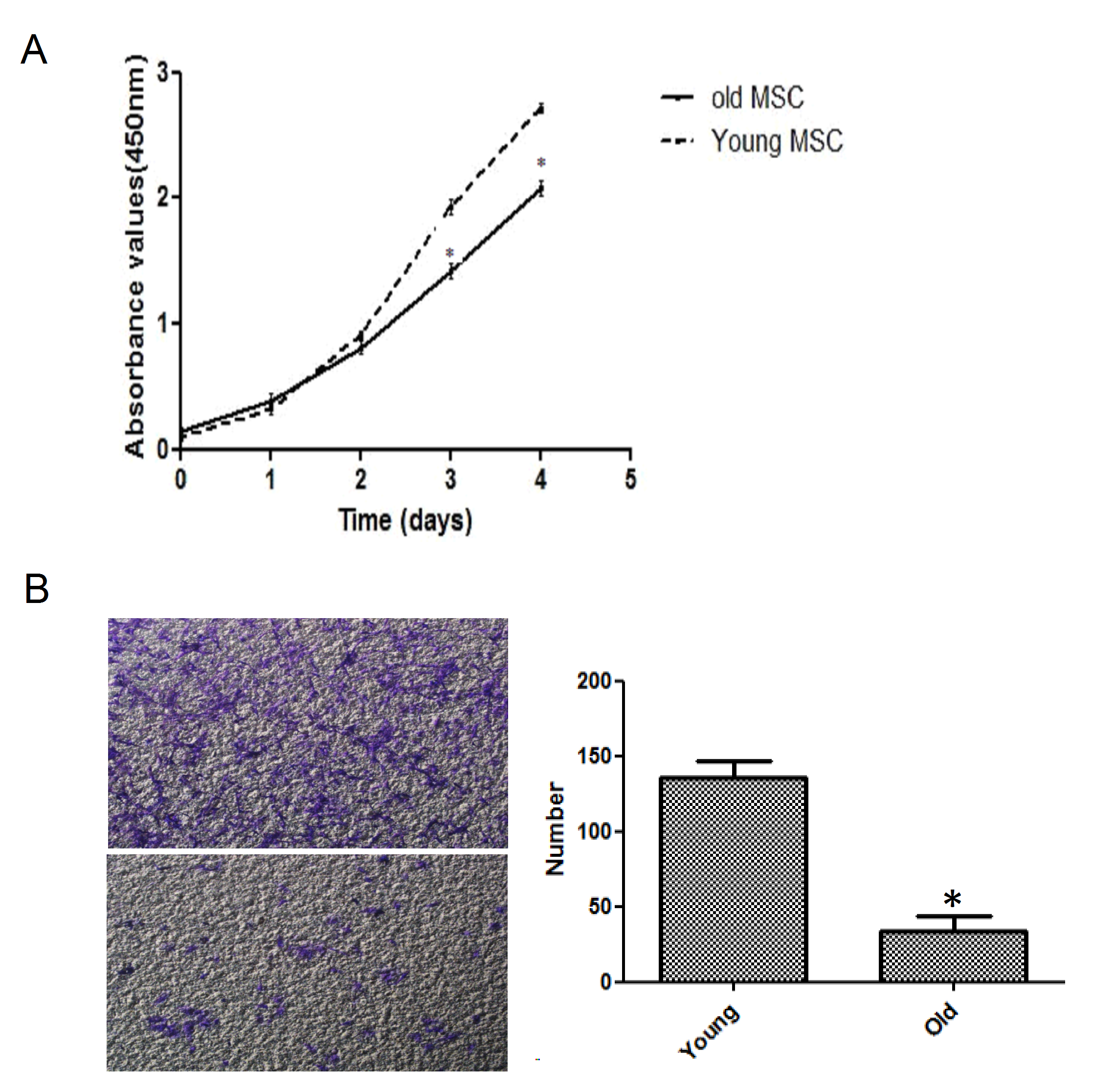


**Additional file 3 The proliferation and migration ability of young and old MSCs.** **(A)** CCK-8 assay shows that the proliferation of the MSCs was not significantly different between the young and old MSCs on days 1 and 2. The absorbance value in the old MSCs was significantly lower than that in the young MSCs on days 3 and 4. **(B)** The number of transferred MSCs in the young group (top) was significantly higher than that in the old group (bottom). (*P<0.05; n = 5)
